# Regulation of androgen receptor splice variant AR3 by PCGEM1

**DOI:** 10.18632/oncotarget.7139

**Published:** 2016-02-02

**Authors:** Ziqiang Zhang, Nanjiang Zhou, Jianguo Huang, Tsui-Ting Ho, Zhuxian Zhu, Zhongmin Qiu, Xinchun Zhou, Chunxue Bai, Fangting Wu, Min Xu, Yin-Yuan Mo

**Affiliations:** ^1^ Department of Pharmacology/Toxicology and Cancer Institute, University of Mississippi Medical Center, Jackson, MS, USA; ^2^ Department of Pulmonary Medicine, Tongji Hospital, Tongji University, Shanghai, China; ^3^ Department of Biochemistry and Cancer Institute, University of Mississippi Medical Center, Jackson, MS, USA; ^4^ Department of Nephrology, Tongji Hospital, Tongji University, Shanghai, China; ^5^ Department of Pathology, University of Mississippi Medical Center, Jackson, MS, USA; ^6^ Department of Pulmonary Medicine, Zhongshan Hospital, Fudan University, Shanghai, PR China; ^7^ System Biosciences, Mountain View, CA, USA; ^8^ Department of Gastroenterology, Affiliated Hospital of Jiangsu University, Zhenjiang, Jiangsu, China

**Keywords:** PCGEM1, androgen receptor, AR3, castration resistance, lncRNA

## Abstract

The androgen receptor (AR) is required for prostate development and is also a major driver of prostate cancer pathogenesis. Thus androgen deprivation therapy (ADT) is the mainstay of treatment for advanced prostate cancer. However, castration resistance due to expression of constitutively active AR splice variants is a significant challenge to prostate cancer therapy; little is known why effectiveness of ADT can only last for a relatively short time. In the present study, we show that PCGEM1 interacts with splicing factors heterogeneous nuclear ribonucleoprotein (hnRNP) A1 and U2AF65, as determined by RNA precipitation and Western blot, suggesting a role for PCGEM1 in alternative splicing. In support of this possibility, PCGEM1 is correlated with AR3, a predominant and clinically important form of AR splice variants in prostate cancer. Moreover, androgen deprivation (AD) induces PCGEM1 and causes its accumulation in nuclear speckles. Finally, we show that the AD-induced PCGEM1 regulates the competition between hnRNP A1 and U2AF65 for AR pre-mRNA. AD promotes PCGEM1 to interact with both hnRNP A1 and U2AF65 with different consequences. While the interaction of PCGEM1 with hnRNP A1 suppresses AR3 by exon skipping, its interaction with U2AF65 promotes AR3 by exonization. Together, we demonstrate an AD-mediated AR3 expression involving PCGEM1 and splicing factors.

## INTRODUCTION

Prostate cancer is the most common malignancy among elderly men in Western countries. The androgen receptor (AR) is a nuclear receptor transcription factor required for normal prostate development and prostate cancer pathogenesis. Furthermore, AR serves as an important therapeutic target. For instance, androgen deprivation therapy (ADT) has been the frontline therapy for treatment of advanced prostate cancer. Although initial response of prostate cancer to ADT is effective, these patients inevitably develop the resistance, i.e., castration-resistant prostate cancer (CRPC) [1, 2]. This is a major obstacle for improving overall survival in prostate cancer. Although this ADT-induced castration resistance has been known for a long time, the underlying mechanism is still elusive. AR is subject to extensive alternative splicing. At least 7 AR splice variants have been identified so far [3, 4]. Among them, AR3 (AR-V7) is one of the major AR splice variants which can play a significant role in castration resistance [5, 6]. More recently, it has been shown that circulating AR3 is associated with the resistance to two clinically important drugs enzalutamide and abiraterone [7].

Alternative splicing represents an important mechanism of genetic diversity in eukaryotes. In this regard, the vast majority of eukaryotic genes including protein-coding genes and long non-coding RNAs (lncRNAs) can be expressed as various alternative splice variants [8] and they may occur in a tissue-specific manner and/or under specific cellular conditions. RNA splicing takes place in a spliceosome, a large and complex molecular machine containing small nuclear ribonucleic particles (snRNPs). Selection of correct splice sites is critical in pre-mRNA splicing and this can be often regulated by various factors. In addition to spliceosome, positive/negative signals such as splicing enhancer/silencer elements in an exon and/or its flanking introns are required for efficient exon recognition, particularly when the exon is alternatively spliced [9, 10]. For example, U2AF65 can bind to exonic splicing enhancer (ESE) or intronic splicing enhancer (ISE) elements to promote exonization. In contrast, heterogeneous nuclear ribonucleoproteins (hnRNPs) such as hnRNP A1 [11] and hnRNP C [12] can bind to exonic splicing silencer (ESS) or intronic splicing silencer (ISS) elements to suppress exonization. However, it remains to be determined which molecular player(s) regulates those interactions to select splice sites in response to environmental cues.

LncRNAs are a large group of poorly characterized non-coding RNAs with >200 nucleotides in length [13]. Accumulating evidence suggests that lncRNAs can play a critical role in regulation of gene expression through various mechanisms [14-16]. Like protein-coding genes, lncRNAs can function as oncogenic and tumor-suppressor genes, thus impacting one or more of the cancer hallmarks. PCGEM1 was identified as a prostate cancer specific lncRNA [17] that is capable of promoting proliferation and inhibiting apoptosis. However, it remains to be determined whether PCGEM1 can regulate AR alternative splicing in response to ADT, leading to castration resistance.

In the present study, we show that androgen deprivation (AD) induces PCGEM1 expression and causes its subcellular re-distribution. Moreover, PCGEM1 functionally interacts with splicing factors including hnRNP A1 and U2AF65. Of considerable interest, the PCGEM1-hnRNP A1 interaction suppresses binding of hnRNP A1 to AR pre-mRNA whereas the interaction of PCGEM1 with U2AF65 enhances its binding to AR pre-mRNA, leading to expression of AR3.

## RESULTS

### PCGEM1 interacts with splicing factors hnRNP A1 and U2AF65

**Figure 1:**
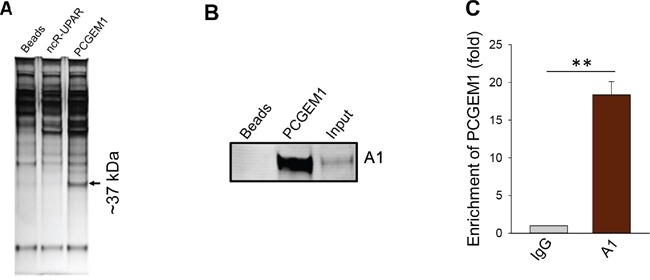
Identification of hnRNP A1 as a PCGEM1 binding partner **A.** A representative silver-stained gel picture showing a ∼37 kDa band specific to PCGEM1 probe. A biotin-labeled PCGEM1 RNA probe was used for the pulldown assay; mass spectrometry analysis indicated that this 37 kDa band is hnRNP A1. ncR-UPAR serves as a negative control. **B.** Verification of the interaction of PCGEM1 with hnRNP A1 by RNA precipitation and Western. Samples were prepared the same way as in Fig. 1A and the membrane was probed with hnRNP A1 antibody. **C.** Detection of PCGEM1 by qRT-PCR after immunoprecipitation with the hnRNP A1 antibody.

PCGEM1 is prostate cancer specific lncRNA [17]. A recent report showed that PCGEM1 and PRNCR1 interact with AR, impacting AR regulated gene expression [18]. However, this may not fully explain why PCGEM1 can promote castration resistance because PCGEM1 can interact with both full-length AR and AR3 [18]. Therefore, we performed RNA precipitation experiments using the biotin-labelled PCGEM1 RNA probe to identify PCGEM1 binding partners. This approach combined with PAGE analysis and mass-spectrometry analysis suggested that heterogeneous nuclear ribonucleoprotein A1 (hnRNP A1) was a potential PCGEM1 binding partner (Fig. 1A; Fig. S1). Subsequent Western blot using hnRNP A1 antibody confirmed this interaction (Fig. 1B). Moreover, RNA immunoprecipitation (RIP) using hnRNP A1 antibody detected a 15-fold enrichment of PCGEM1 over IgG (Fig. 1C). Since hnRNP A1 is a splicing factor, the interaction of PCGEM1 with hnRNP A1 may suggest a role for PCGEM1 in AR splicing.

### Upregulation of PCGEM1 is associated with AR3 expression and castration resistance in prostate cancer cell lines

**Figure 2:**
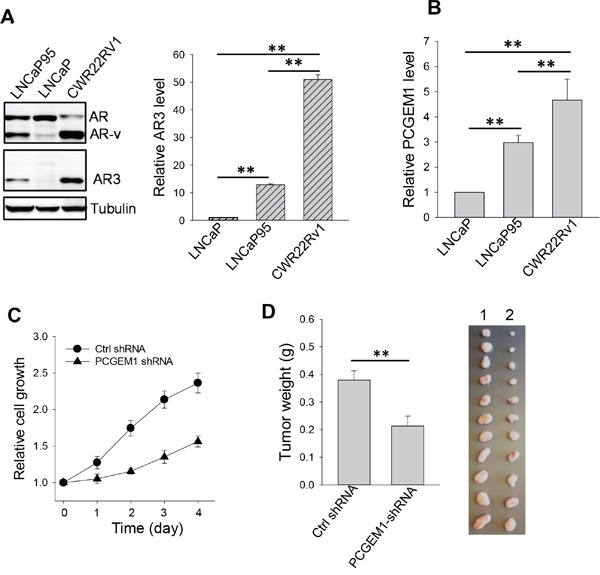
Effect of PCGEM1 on castration resistance **A.** Relative expression of AR, AR variants (AR-v) and AR3 in LNCaP, LNCaP95 and CWR22Rv1 cells, as detected by Western (left) using N-terminal AR antibody or AR3 specific antibody; and AR3 mRNA levels, as detected by qRT-PCR (right) using AR3 specific primers (AR3-RT-5.1 and AR3-RT-3.1). β-actin was used as an internal control. **B.** Expression of PCGEM1 by qRT-PCR in LNCaP, LNCaP95 and CWR22Rv1 cells. **C.** PCGEM1-shRNA suppresses CWR22Rv1 cell growth. The cells were grown in androgen-free medium and the cell growth was measured for 4 days by MTT. **D.** PCGEM1-shRNA reduces tumor growth in the xenograft mouse model. CWR22Rv1 cells were injected into castrated male SCID mice subcutaneously as described in the text, and tumor growth was measured 7 days after injection. Tumors were harvested and weighted at day 31 after injection of tumor cells. 1, Ctrl shRNA; 2, PCGEM1-shRNA. Values in A, B and D are means ± SE (n = 3). **, p < 0.01, two-sided two-sample t test.

It is well known that AR splice variants play a significant role in castration resistance [5, 6]. Next, we determined the role of PCGEM1 in regulation of AR splicing and castration resistance. In this regard, we found a positive correlation of PCGEM1 with AR3, a major splice variant [19], in prostate cancer cell lines. For example, androgen sensitive LNCaP cells expressed the full-length AR whereas AR3 was barely detectable (Fig. 2A). In contrast, castration resistant LNCaP95 and CWR22Rv1 cells expressed both AR3 and the full-length AR. LNCaP95 is an androgen-independent cell line derived from long-term continuous culture of LNCaP cells in androgen-depleted conditions [6]; CWR22Rv1 was derived from xenograft tumors that were serially propagated in mice after castration-induced regression and relapse of the parental androgen-dependent CWR22 xenograft [20]. Apparently, the AR3 level was higher in CWR22Rv1 than in LNCaP95 cells (Fig. 2A). This trend was also seen at the AR3 mRNA level in these cell lines (Fig. 2A, right). Of interest, the PCGEM1 level was also higher in the resistant cell lines CWR22Rv1 and LNCaP95 than in the sensitive LNCaP cells (Fig. 2B). We next determined whether PCGEM1 can promote castration resistance. MTT assays revealed that suppression of PCGEM1 by RNAi sensitized CWR22Rv1 cells to AD (Fig. 2C). Furthermore, suppression of PCGEM1 significantly reduced tumor growth (Fig. S2) and the tumor weight in castrated male mice (Fig. 2D). Together, these results suggest that PCGEM1 is able to promote castration resistance both *in vitro* and *in vivo* possibly through regulation of AR3 expression.

### Androgen deprivation upregulates PCGEM1 and causes its subcellular re-distribution

**Figure 3:**
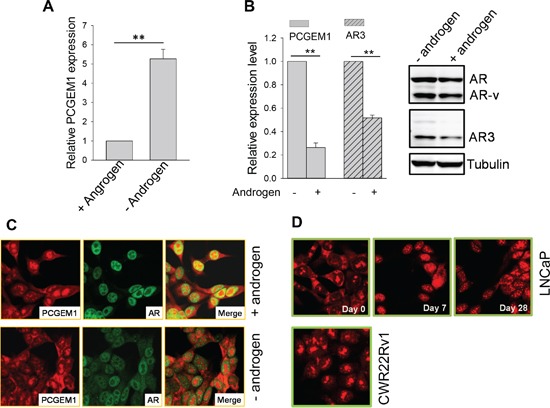
Androgen deprivation induces PCGEM1 expression and its subcellular re-localization **A.** AD induces PCGEM1 in LNCaP cells. The cells were cultured in the presence or absence of androgen for 7 days before harvesting for qRT-PCR. **B.** Reversible regulation of PCGEM1 and AR3 in LNCaP95 cells in response to AD. The cells that were originally maintained in androgen-free were switched to medium supplemented with 10 nM R1881 and grown for 2 days. The cells were then harvested for qRT-PCR (left) to detect PCGEM1 and AR3 RNA or Western (right) to detect AR3 at the protein level. **C.** Subcellular distribution of PCGEM1 in response to androgen. PCGEM1 was detected by FISH and AR was detected by IF. LNCaP cells were grown in androgen containing medium (top) or in androgen free medium for 2 days (bottom) before fixation for FISH and IF. **D.** Subcellular distribution of PCGEM1 in response to AD. Cells were fixed and PCGEM1 was detected by FISH. Only day 0 was shown for CWR22Rv1 because the distribution of PCGEM1 was same from day 0, day7 and day 28. Values in A and B are means ± SE (n = 3). **, p < 0.01, two-sided two-sample t test.

Given the positive relationship between PCGEM1 and AR3, we decided to determine whether PCGEM1 specifically impacts AR3 expression, leading to castration resistance. We found that PCGEM1 was significantly induced in LNCaP cells by AD (Fig. 3A). This AD-induced PCGEM1 was dependent on AR status because such induction was not seen in AR negative PC3 and DU-145 cells (Fig. S3). Of interest, levels of PCGEM1 and AR3 are reversible depending on androgen in LNCaP95 cells. For example, PCGEM1 and AR3 (both RNA and protein) were significantly decreased when the cells were put back in androgen-containing medium (Fig. 3B). Furthermore, FISH assays revealed subcellular re-localization of PCGEM1 in LNCaP cells in response to AD. In the presence of androgen, PCGEM1 was detected in both cytoplasm and nucleus (Fig. 3C, top). This signal was specific to PCGEM1 because the blocking oligo complementary to the probe was able to completely abolish the signal (Fig. S4). As a control, AR was exclusively in the nucleus as expected (Fig. 3C). In the absence of androgen for 2 days, there were more PCGEM1-containing nuclear speckles (Fig. 3C, bottom).

Nuclear speckles are important to RNA splicing and gene expression [21]. To better characterize the subcellular re-distribution of PCGEM1 in response to AD, we grew LNCaP cells in androgen-free medium for up to 28 days. Starting from day 7, we observed the majority PCGEM1 signal in the nucleus, particularly nuclear speckles (Fig. 3D, top). However, AD had little effect on PCGEM1 subcellular localization in CWR22Rv1 cells during 28 days of AD (Fig. 3D, bottom).

### hnRNP A1 is a repressor for AR3 expression

**Figure 4:**
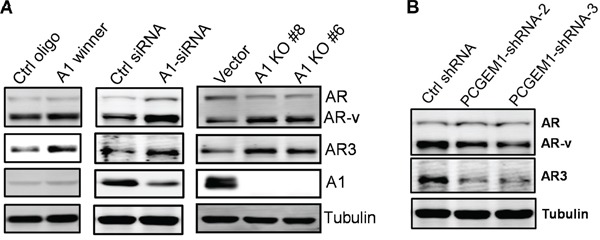
Regulation of AR3 by hnRNP A1 and PCGEM1 **A.** Increasing AR3 expression by A1 winner, hnRNP A1-siRNA or hnRNP A1 knockout. CWR22Rv1 cells were transiently transfected with A1 winner oligo or hnRNP A1-siRNAs and were then harvested 2 days later for Western. hnRNP A1 knockout was made through CRISPR/Cas9 as described in the text. **B.** Suppression of PCGEM1 by RNAi reduces AR3 expression in CWR22Rv1 cells.

Since hnRNP A1 is well-known for its role in mRNA processing or alternative splicing [22, 23], and it may function as a splicing silencer factor [24], we determined whether hnRNP A1 is a negative regulator for AR3 expression. In support of this notion, we identified two putative hnRNP A1 binding sites [25] in the intron between exon 3 (E3) and exon 3b (E3b) (Fig. S5A). Through screening of randomized RNA oligos against hnRNP A1, Burd and Dreyfuss identified an hnRNP A1 winner sequence (UAUGAUAGGGACUUAGGGUG) with two putative binding sites (underlined) [25]. As expected, this A1 winner oligo was able to increase AR3 expression in CWR22Rv1 cells as compared to control oligo (Fig. 4A, left). Moreover, hnRNP A1 siRNA (Fig. 4A, middle) or knockout (Fig. 4A, right) also enhanced AR3 expression in CWR22Rv1 cells, further supporting a suppressive role of hnRNP A1 in AR3 expression. In contrast, PCGEM1 shRNAs suppressed AR3 expression (Fig. 4B).

### U2AF65 is an enhancer for AR3 expression

**Figure 5:**
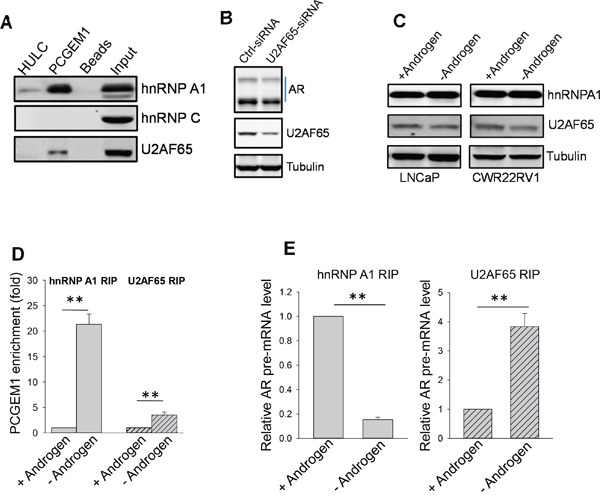
PCGEM1-hnRNP A1 interaction suppresses, whereas PCGEM1-U2AF65 interaction enhances AR3 expression **A.** PCGEM1 interacts with hnRNP A1 and U2AF65, but not hnRNP C, as determined by RNA precipitation and Western. **B.** Suppression of U2AF causes reduction of AR variants (AR-v). **C.** No difference is seen for hnRNP A1 or U2AF expression between LNCaP and CWR22Rv1 cells in the presence or absence of androgen. **D.** AD enhances the interaction of PCGEM1 with hnRNP A1 and U2AF. **E.** While the interaction of hnRNP A1 with AR pre-mRNA is decreased after AD, the interaction of U2AF65 with AR pre-mRNA is increased. Values in D and E are means ± SE (n = 3). **, p < 0.01, two-sided two-sample t test.

Although other members of hnRNP family such as hnRNP C have been implicated in suppression of alternative splicing of Alu elements [12], RNA precipitation with PCGEM1 probe revealed that PCGEM1 did not interact with hnRNP C (Fig. 5A), suggesting that PCGEM1 specifically interacts with hnRNP A1. However, this assay identified an additional splicing factor U2AF65 that interacted with PCGEM1 (Fig. 5A). U2AF65 is capable of binding to poly-pyrimidine track region of pre-mRNA and it functions as a splicing enhancer factor [26]; In particular, there is a putative U2AF65 binding site (UCUCUCUUUC) in the 3′ end of the intron (Fig. S5A). U2AF65-siRNA suppressed AR3 expression (Fig. 5B), suggesting the importance of U2AF65 in AR3 expression. However, expression levels of hnRNP A1 or U2AF65 were not affected by AD (Fig. 5C). In contrast, AD not only induced PCGEM1 expression (Fig. 3A), but also facilitated the interaction of PCGEM1 with hnRNP A1 and U2AF65, as detected by RIP (Fig. 5D). Of particular interest, such interactions had very different consequences. The increased PCGEM1-hnRNP A1 interaction caused a significant decrease in the ability of hnRNP A1 to interact with AR pre-mRNA (Fig. 5E, left). In contrast, the increased PCGEM1-U2AF65 interaction promoted its interaction with AR pre-mRNA (Fig. 5E, right), suggesting that along with PCGEM1, hnRNP A1 is capable of suppressing AR3 whereas U2AF65 is capable of promoting AR3.

### Androgen deprivation promotes the co-localization of PCGEM1 with U2AF65 in nuclear speckles

**Figure 6:**
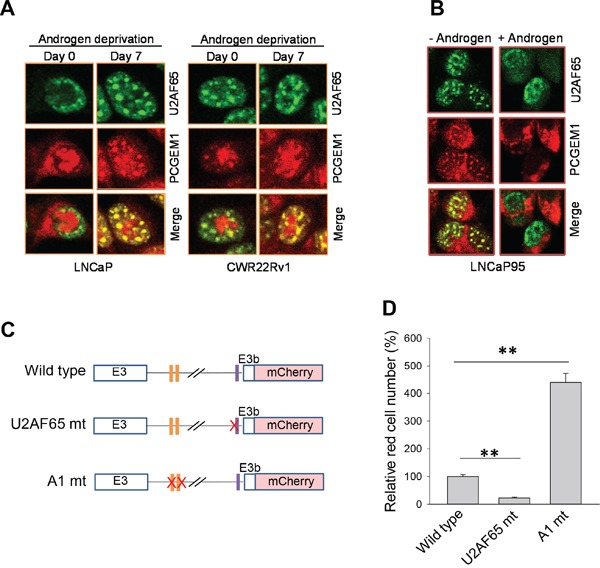
Co-localization of PCGEM1 and U2AF65 in nuclear speckles and the role of the binding sites of hnRNP A1 and U2AF65 in AR3 expression **A.** U2AF65 and PCGEM1 are co-localized in nuclear speckles in response to AD in LNCaP cells (left). However, AD has no effect on this co-localization in CWR22Rv1 cells (right). **B.** Reversible nuclear speckle localization of PCGEM1 in LNCaP95 cells in response to androgen. The cells were maintained in androgen-free medium and then switched to androgen-containing medium for 2 days. **C & D.** Binding sites for hnRNP A1 and U2AF65 in AR pre-mRNA are important to AR3 expression. CWR22Rv1 cells were transfected with mini-gene cassette reporters as indicated (C) and mCherry signals were examined 24 h after transfection. Relative expression of mCherry signals by randomly examining 10 fields and then normalized as 100% for AR3 wild type reporter. Values in (D) are means ± SE (n = 3). **, p < 0.01, two-sided two-sample t test.

Nuclear speckles have been shown to be critical sites for RNA processing [21]. AD caused accumulation of PCGEM1 in the nuclear speckles (Fig. 3D) in addition to upregulation of the PCGEM1 level (Fig. 3A). Before AD, a relatively low level of U2AF65 was detected in nuclear speckles. Under AD for 7 days, more U2AF65 was accumulated in the nuclear speckles in LNCaP cells (Fig. 6A). Similarly, a fair amount of PCGEM1 was found in these nuclear speckles, co-localizing with U2AF65 (Fig. 6A, left). In CWR22Rv1 cells, androgen had little effect on the subcellular localization of PCGEM1 or U2AF65 (Fig. 6A, right). Of interest, this androgen-mediated redistribution of PCGEM1 and U2AF65 was also reversible in LNCaP95 cells. For example, upon addition of androgen to the medium, nuclear speckle staining for both PCGEM1 and U2AF65 remarkably decreased (Fig. 6B).

### The binding sites of hnRNP 1 and U2AF65 are required for expression of AR3 reporters

To further determine the role of PCGEM1 in AR3 expression, we generated a mini-gene cassette reporter carrying mCherry (Fig. S5A). As expected, mCherry signal was higher in CWR22Rv1 cells than in LNCaP cells (Fig. S5B). Ectopic expression of PCGEM1 increased the signal in LNCaP cells (Fig. S5C). To determine the role of the hnRNP A1 and U2AF65 binding sites in AR3 expression, we mutated these sites separately (Fig. 6C). As shown in Fig. 6D and Fig. S6, mutation of hnRNP A1 binding sites at AR pre-mRNA increased the mCherry signal. In contrast, mutation of the U2AF65 binding site abolished the signal. These results are consistent with the finding that interactions of PCGEM1-hnRNP A1 and PCGEM1-U2AF65 have different consequences (Fig. 5D-5E).

## DISCUSSION

The present study demonstrates that AD induces PCGEM1 and causes its localization of nuclear speckles. Through interaction with splicing factors, such as hnRNP A1 and U2AF65, PCGEM1 promotes AR3 expression. As a major AR splice variant, AR3 has been shown to play a significant role in castration resistance [27] and thus identification of this AD-PCGEM1-AR3 axis may explain in part why the effectiveness of ADT can only last for a relatively short time. Our study suggests that PCGEM1 participates in the AR signaling by regulation of AR splice variants in response to AD. Moreover, our *in vitro* and xenograft assays with castrated male mice support the role of PCGEM1 in castration resistance. Together, these findings highlight the importance of PCGEM1 in AR signaling and castration resistance.

As components of the basic machinery for RNA splicing, hnRNP A1 and U2AF65 are important to AR3 expression although AD has no effect on their expression levels. The hnRNP proteins belong to the RNA binding protein family that play multiple functions. Along with other RNP proteins, they participate in pre-mRNA processing such as splicing, and are important determinants of mRNA export, localization, translation, and stability [28]. Several members of this family have been implicated in alternative splicing. In this regard, it appears that a specific member of these proteins may be responsible for a specific set of gene splicing. For instance, hnRNP C can compete with U2AF65 to specifically suppress splicing of Alu exons [12]. We show that hnRNP C does not interact with PCGEM1. This may be attributed to the ability of hnRNP C to preferably bind poly uridine regions [12]. In contrast, hnRNP A1 tends to bind relatively purine-rich regions (e.g., UAGGGA/U). This finding is consistent with the report that hnRNP C has little overlap with hnRNP A1 or other members of hnRNP protein except for hnRNP I which has a slight overlap [12]. Of interest, genetic mutations at hnRNP C binding sites play a major role in exonization of Alu exon, leading to various diseases. Thus, it is possible that PCGME1 may play a similar role. AD-induced PCGEM1 and its subcellular redistribution provide more flexibility of AR3 regulation. Therefore, like genetic mutations, environmental cue offers additional mechanisms for alternative splicing.

Regulation of alternative splicing is a complex process. It is known that splicing factors select splice sites often in a concentration-dependent manner and thus, the relative expression of these factors may decide a particular splice pattern [29]. The present study suggests that this can be achieved by AD-mediated PCGEM1. For instance, AD increases the interaction of PCGEM1 with hnRNP A1 and U2AF65 (Fig. 5D). Once bound by PCGEM1, the binding activity of hnRNP A1 to AR pre-mRNA is reduced; on the other hand, the binding activity of U2AF65 to AR pre-mRNA is increased. In support of this notion, we show that AD promotes the localization of U2AF65 in nuclear speckles (Fig. 6A). In particular, this redistribution seems to be closely associated with PCGEM1. However, in resistant CWR22Rv1 cells, such redistribution of U2AF65 is not obvious, which may be explained by the possibility that CWR22Rv1 cells preferably form a PCGEM1-U2AF65-AR pre-mRNA complex, leading to constitutive expression of AR3.

However, the detail mechanism still remains to be determined as to why the PCGEM1-hnRNP A1 interaction causes the loss of its suppressive function whereas the PCGEM1-U2AF65 interaction promotes the binding of U2AF65 to AR pre-mRNA, leading to expression of AR3. We speculate at least two possibilities for this opposing role of PCGEM1 once bound by these splicing factors. Although hnRNP A1 is a RNA binding protein, interacting with many types of RNAs, several reports suggest that hnRNP A1 prefer the UAG motif [25, 30]. For instance, screening randomized oligos identified several oligos that are preferably bound by hnRNP A1 and among them A1 winner oligo is the top preferable site [25]. There are two conserved A1 binding in AR intron between E3 and E3b. We show that these two binding sites are important because mutation of these two sites causes a significant increased mCherry signal. On the other hand, although PCGEM1 does not carry these conserved binding sites, there are over 10 UAG motifs through which PCGEM1 may compete with AR pre-mRNA for hnRNP A1. Our RIP assay with hnRNP A1 antibody supports this possibility. Another possibility is that binding of PCGEM1 to U2AF65 may enhance its competition with hnRNP A1 for AR pre-mRNA. Once bound by PCGEM1, U2AF65 may become more competitive. Through the similar mechanism, PCGEM1 might also be involved in regulation of other AR splice variants.

PCGEM1 is an interesting molecule, but the role of PCGEM1 in prostate cancer is still controversial. For example, it has been reported that PCGEM1 along with PRNCR1 can impact AR signaling through interaction with AR to promote castration resistance [18], however, a comprehensive analysis of RNA-sequencing data (RNA-seq) does not support this notion [31]. Similarly, a recent study suggests that PCGEM1 is stimulated by androgen and downregulated by castration in xenograft models [32], which is inconsistent with our findings. Furthermore, their study showed no subcellular distribution of PCGEM1 in response to AD. Although this might be due to different systems used in these studies, such a controversy will certainly stimulate further investigations.

In summary, our study suggests that the interaction of PCGEM1 with splicing factors such as hnRNP A1 and U2AF65 determines the fate of AR3 (Fig. S7). One function of hnRNP A1 is to interact with AR pre-mRNA at hnRNP A1 binding sites, which subsequently inhibits the binding to AR pre-mRNA by splicing enhancers such as U2AF65 under normal physiological conditions. However, AD causes upregulation of PCGEM1 (Fig. 3A) and increases the accumulation of PCGEM1 in nuclear speckles (Fig. 3D). Thus, more PCGEM1 interacts with hnRNP A1 and U2AF65 (Fig. 5D), which, however, can have opposite consequences. Once bound by PCGEM1, hnRNP A1 is no long able to interact with AR pre-mRNA (Fig. 5E) to suppress U2AF65 binding to AR pre-mRNA. On the other hand, the ability of the PCGEM1-bound U2AF65 to interact with AR pre-mRNA is increased, facilitating their co-localization in nuclear speckles (Fig. 5E) and promoting AR3 expression and castration resistance.

## MATERIALS AND METHODS

### Cell culture

Prostate cancer LNCaP and CWR22Rv1 cells were purchased from ATCC; LNCaP95 was a generous gift from Dr. Alan K. Meeker, Johns Hopkins University School of Medicine. Cells were grown in phenol free RPMI 1640 (Lonza, Walkersville, MD) supplemented with charcoal stripped 5% FBS (Sigma-Aldrich). HECK293T cells were grown in DMEM supplemented with charcoal stripped 10% FBS. All media were supplemented with 2 mM glutamine, 100 units of penicillin/ml, and 100 μg of streptomycin/ml (Lonza). Cells were incubated at 37°C and supplemented with 5% CO_2_ in the humidified chamber.

### Lentivirus preparation and infection

Lentiviral packaging was carried out in HEK-293T cells using a packaging system from SBI per the manufacturer's protocol, as described previously [33]. For infection, exponentially growing cells were mixed with viral particles in the presence of polybrene (0.8 mg/ml) in a six-well plate at a multiplicity of infection of 1∼3.

### RNA precipitation

To determine which proteins are associated with PCGEM1, we performed RNA precipitation assay using synthesized PCGEM1 as a probe. In brief, the DNA fragment covering the entire PCGEM1 sequence was amplified by PCR using a T7 containing primers (T7-PCGEM1-5.1 and T7-PCGEM1-Not1-3.1), and then cloned into pCR8 (Life Technology). In addition, two lncRNAs, ncR-UPAR and HULC, were also cloned in the same way as negative controls. The resultant plasmid DNA was linearized with restriction enzyme Not I which was introduced from the reverse PCR primer, and then used to synthesize RNA by T7 polymerase. A 20 μl reaction contained 400 ng linearized plasmid DNA, 20 U ribonuclease inhibitor, 2.5 mM NTP mixture supplemented with 10% biotin labeled UTP (Perkin Elmer) and 20 U T7 RNA polymerase (New England BioLabs); and then it was incubated at 37°C for 60 min, followed by 25 U RNase-free DNase I (New England BioLabs) at 37°C for 30 min. The labeled RNA was purified by a column-based kit (Zymo Research). Cellular extract was prepared from a 10 cm dish culture (∼80% confluence) with cell lysis buffer [34]. For precipitation assays, the reaction (RNA probe and cellular extract) was incubated at 4°C for 60 min, followed by 5 washes with PBS. The pellets were used either for extraction of RNA for RT-PCR or for Western according to standard procedures.

### RNA immunoprecipitation (RIP)

To determine the interaction of hnRNP A1 or U2AF65 with PCGEM1 and AR pre-mRNA, we used hnRNP A1 or U2AF65 antibody for pulldown assays and then detected PCGEM1 or AR pre-mRNA by qRT-PCR using specific primers listed in Supplementary Table 1. Magna RIP™ RNA-Binding Protein Immunoprecipitation Kit (Millipore) was used for RIP procedures according to the manufacturer's protocol. After the antibody was recovered by protein A+G beads, standard qRT-PCR was performed to detect RNA levels in the precipitates.

### Fluorescence *in situ* hybridization (FISH)

FISH was used to detect PCGEM1 levels in prostate cancer cell lines. Biotin-labeled antisense LNA probes derived from PCGEM1 were listed in Supplementary Table 1. The procedure was essential as previously described [35] except that signals were revealed by TSA™ Kit #24 with Alexa Fluor 568 (Life Technology).

### Immunofluoresence staining

Immunofluoresence staining was used to detect AR, hnRNP A1 and U2AF65, as described previously [34]. In brief, cells were 3% paraformaldehyde and permeablized by 80% cold methanol. After washing with PBS (phosphate buffered saline), coverslips were then incubated in PBS with 3% BSA for 10 min at room temperature. Primary antibodies against individual protein in PBST (PBS plus 0.1% Tween 20) were then added and incubated for 1 h at room temperature. After 3 washes with PBS, the cells were incubated with a fluorescence-conjugated secondary antibody conjugated with either Alexa Fluor 568 or Alexa Fluor 488 in the dark for 1 h. For nuclear staining, the cells were subsequently stained in 0.5 μg/ml Hoechst dye for 5 min before examinations under a fluorescence microscope.

### MTT assay

MTT assay was performed to determine the effect of PCGEM1 knockdown on cell growth as described previously [34]. Infected CWR22Rv1 cells carrying control shRNA or PCGEM1-siRNAs were grown in regular medium and the relative cell growth was daily measured from day 0 to day 4.

### Xenograft mouse model

The animal studies were conducted in accordance with NIH animal use guidelines and the experimental protocol was approved by the UMMC's Animal Care and Use Committee. Male SCID mice at 5∼6 week old were first castrated and one week later infected CWR22Rv1 cells (control shRNA or PCGEM1-shRNAs) were then injected subcutaneously into these mice with 1 million cells containing 50% matrigel per spot, two spots per animal and 6 animals per group. Tumor growth was monitored every other day and harvested at day 31 after injection. The two-group t test was used to compare two means at each time point. All animals were included for analysis.

### Knockout of hnRNP A1 by CRISPR/Cas9

To facilitate the selection of positive clones resulted from CRISPR/Cas9, we generated a donor vector in such a way that targeting sequence is replaced by marker genes (GFP and PU, the puromycin resistance gene) once it is integrated into the genomic DNA by homologous recombination. Donor vector carried ∼800 bp targeting sequence at each side and EF1-GFP-T2A-PU in the middle, flanked by a LoxP site. The dual gRNA construct carrying Cas9 and donor vector were introduced into CWR22Rv1 cells by transient transfection. One week later, the transfected cells were subject to puromycin selection; and surviving cells were sorted by FACS based on GFP signal into individual wells of 96-well plates and incubated for 2∼3 weeks, as described previously [36]. The sorted clones were then expanded in 24 well plates. Complete knockout clones were identified by genomic PCR and Western.

### Statistical analysis

The two-sample t test was used to compare two mean expressions. Bonferroni correction was used to adjust the p values of pairwise comparisons among three mean expressions. Relationship between PCGEM-1 and AR expression was studied by depicting scatter plot and calculating Pearson correlation coefficient. All p values were two sided and p values less than 0.05 were considered as significant.

## SUPPLEMENTARY MATERIALS AND METHODS, FIGURES AND TABLE





## Abbreviations

AR: androgen receptor
IF: immunofluorescence
FISH: fluorescence *in situ* hybridization
hnRNP: heterogeneous nuclear ribonucleoprotein
lncRNA: long non-coding RNA
PCR: polymerase chain reaction
RT: reverse transcription
RIP: RNA immunoprecipitation
qRT-PCR: quantitative RT-PCR.
