# Supplementary material for: Regulation of androgen receptor splice variant AR3 by PCGEM1
Source: Oncotarget. 2016 Feb 2;7(13):15481–91. doi: 10.18632/oncotarget.7139 (PMC4941255; doi:10.18632/oncotarget.7139)
Supplement: Supplementary file 1 [file oncotarget-07-15481-s001.pdf]

SUPPLEMENTARY MATERIALS AND METHODS

Reagents

Primary antibodies against full-length AR (#5153), and AR3 (#AG10008) were purchased from Cell Signaling and Precision Antibody, respectively; hnRNP A1 (sc-32301), hnRNP C (sc-15386), U2AF65 (sc-48804) from Santa Cruz; GAPDH (10494-1-AP) from ProteinTech, β-actin (A2228) from Sigma. Secondary antibodies conjugated with IRDye 800CW or IRDye 680 were purchased from LI-COR Biosciences (Lincoln, NE). PCGEM1 siRNAs and control siRNA were purchased from Dharmacon or IDT. PCGEM1 shRNAs were from Applied Biological Materials (British Columbia, Canada). Biotin-labeled PCGEM1-LNA probes and control oligos for in site hybridization were purchased from Exiqon (2950 Vedbaek, Denmark). R1881 was purchased from Sigma. PCR primers, A1 winner oligo and blocking oligos were purchased from IDT.

Plasmid construction

PCR reactions for cloning purpose used Phusion enzyme (New England BioLabs). The entire PCGEM1 sequence was amplified by RT-PCR using primers PCGEM1-R1-5.1 and PCGEM1-Not1-3.1, and then cloned into the expression vector pCDH-MSCV-EF1-GFP-T2A-Pu (SBI) using Cold Fusion kit (SBI). The same strategy was used to generate other constructs in this study, otherwise stated.

AR3 mini gene cassette mCherry reporter was constructed as follows. AR E3 and part of intron (2.3 kb) was amplified by PCR from human genomic DNA using primers pCDH-Myc-R1-AR3-E3-5.1 and AR-I3-5.2+I3-3.1-R; intron plus part of E3b (1 kb) was amplified using primers AR-I3-5.2+I3-3.1-R and AR3-mC-3.1; mCherry was amplified using primers AR3-mC-5.1 and mC-pCDH-Sal1-3.1. These three fragments were simultaneously cloned into pCDH-Pu at EcoR I and Not I sites by Cold Fusion kit, as described previously (1) to make a first version of mini-gene reporter. The second version of mini-gene cassette carried two mutations at the first two internal methionines, using the first version as a template and primers AR-I3-Bsu36-5.1 and mC-mut-3.1; mC-mut-5.1 and mC-Sbf1-3.1. The same approach was used to make mutant hnRNP A1 and U2AF65 constructs, with primers AR-E3-R1-5.1/A1-mt-3.1 and A1-mt-5.1/AR-I3-BsrG1-3.1. All PCR products were verified by DNA sequencing.

Transfection

Cells were transfected with siRNAs using RNAfectin reagent (Applied Biological Materials) or plasmid DNA using DNAfectin (Applied Biological Materials) following the manufacturer's protocol.

qRT-PCR

To specifically detect expression of PCGEM1 and AR3, we used the SYBR Green method with primers listed in Table S1. Total RNA was isolated by TRIzol reagent (Life Technology). Reverse transcription was carried out by using RevertAid™ Reverse Transcriptase (ThermoFisher) and random primer mix (New England BioLabs). β-actin or GAPDH was used as an internal control. Delta-delta Ct values were used to determine their relative expression as fold changes, as previously described (2).

Western blot

Cells were harvested, and protein was extracted from transfected cells and quantified as previously described (1) using polyacrylamide gradient SDS gel.

REFERENCE

1. Sachdeva M, Zhu S, Wu F, Wu H, Walia V, Kumar S, et al. p53 represses c-Myc through induction of the tumor suppressor miR-145. Proceedings of the National Academy of Sciences of the United States of America. 2009;106:3207-12. Epub 2009/02/10.
2. Si ML, Zhu S, Wu H, Lu Z, Wu F, Mo YY. miR-21-mediated tumor growth. Oncogene. 2007;26:2799-803. Epub 2006/10/31.

hnRNP A1 amino acid sequences:

MSKSESPKEPEQLRKLFIGGLSFETTDESLRSHFEQWGTLTDCVVMRDPNTKRSRGFGFVTYATVEEVDA
AMNARPHKVDGRVVEPKRAVSREDSQRPGAHLTVKKIFVGGIKEDTEEHHLRDYFEQYGKIEVIEIMTDR
GSGKKRGFAFVTFDDHDSVDKIVIQKYHTVNGHNCEVRKALSKQEMASASSSQRGRSGSGNFGGGRGGGF
GGNDNFGRGGNFSGRGGFGGSRGGGGYGGSGDGYNGFGNDGSNFGGGGSYNDFGNYNNQSSNFGPMKGGN
FGGRSSGPYGGGGQYFAKPRNQGGYGGSSSSSSYGSGRRF

Peptides identified from mass spectrometry analysis aligned with predicted sequences:

DYFEQYGK
EDTEEHHLR
IEVIEIMTDR
SESPKEPEQLR
EDSQRPGAHLTVK
YHTVNGHNCEVR
SSGPYGGGGQYFAKPR
NQGGYGGSSSSSSYGSGR
GFAFVTFDDHDSVDK
LFIGGLSFETTDESLR
IFVGGIKEDTEEHHLR
SHFEQWGTLTDCVVMR
KLFIGGLSFETTDESLR
EDTEEHHLRDYFEQYGK
DYFEQYGKIEVIEIMTDR
GFGFVTYATVEEVDAAMNARPHK
SHFEQWGTLTDCVVMRDPNTK

Blue and red color were used for separation from individual peptides.

Supplementary Figure S1: Identification of hnRNP A1 as PCGEM1 binding partner. Peptide sequences identified by mass spectrometry.

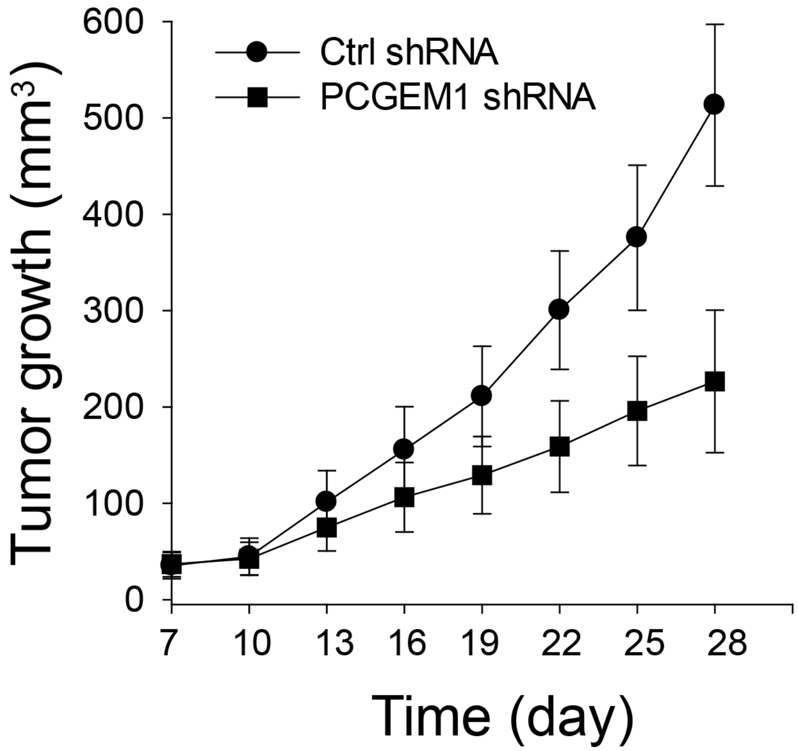

Supplementary Figure S2: PCGEM1-shRNA reduces cell growth in the xenograft mouse model. CWR22Rv1 cells were injected castrated SCID male mice subcutaneously and tumor growth was measured every other day 7 days after injection.

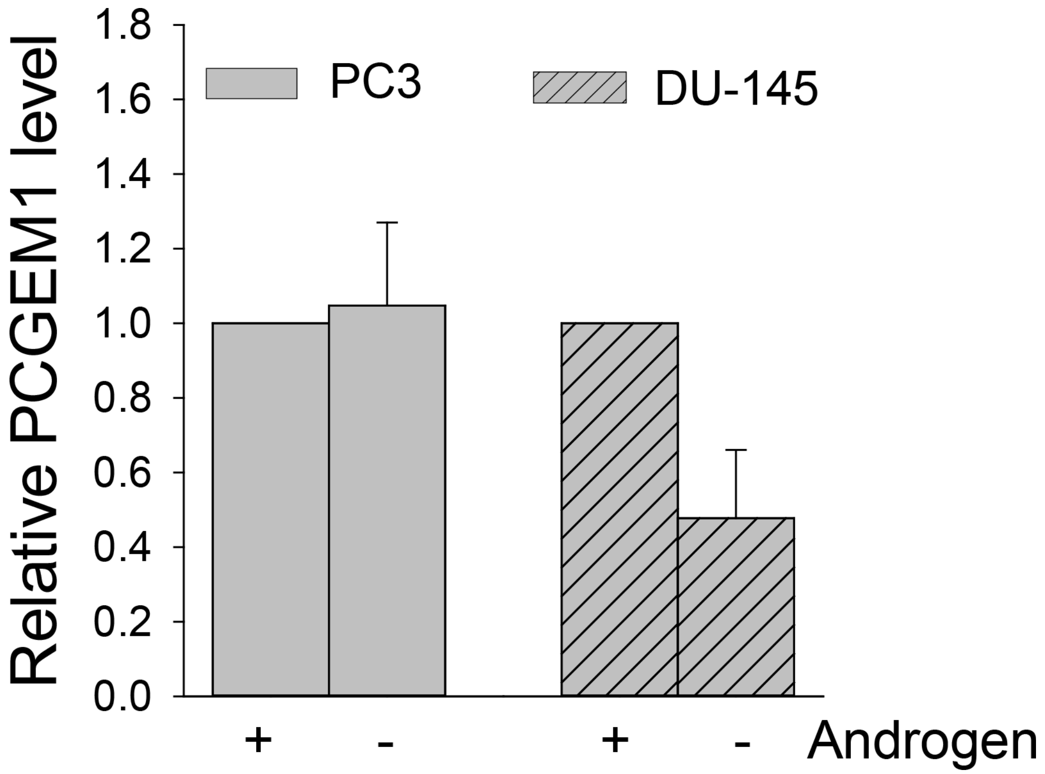

Supplementary Figure S3: Androgen deprivation does not induce PCGEM1 in AR negative PC3 and DU145 cells, as detected by qRT-PCR.

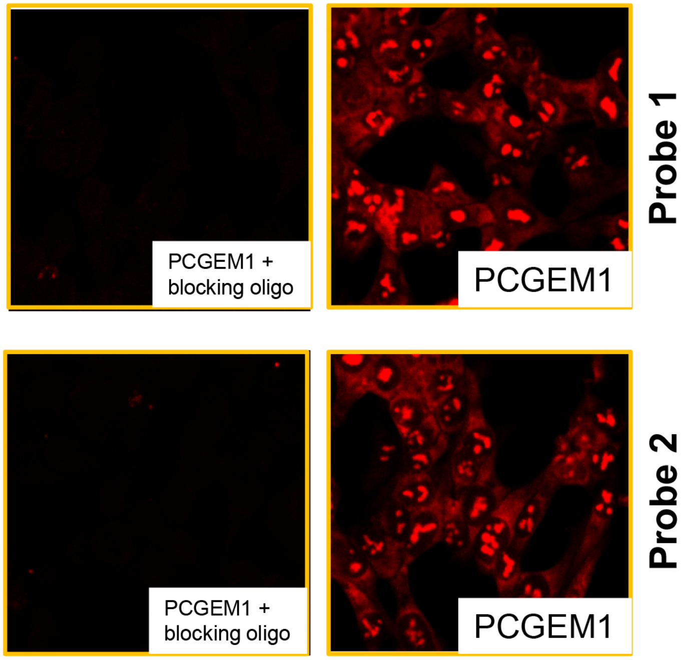

Supplementary Figure S4: Detection of PCGEM1 by fluorescence in situ hybridization (FISH) and the signal was blocked by blocking oligos.

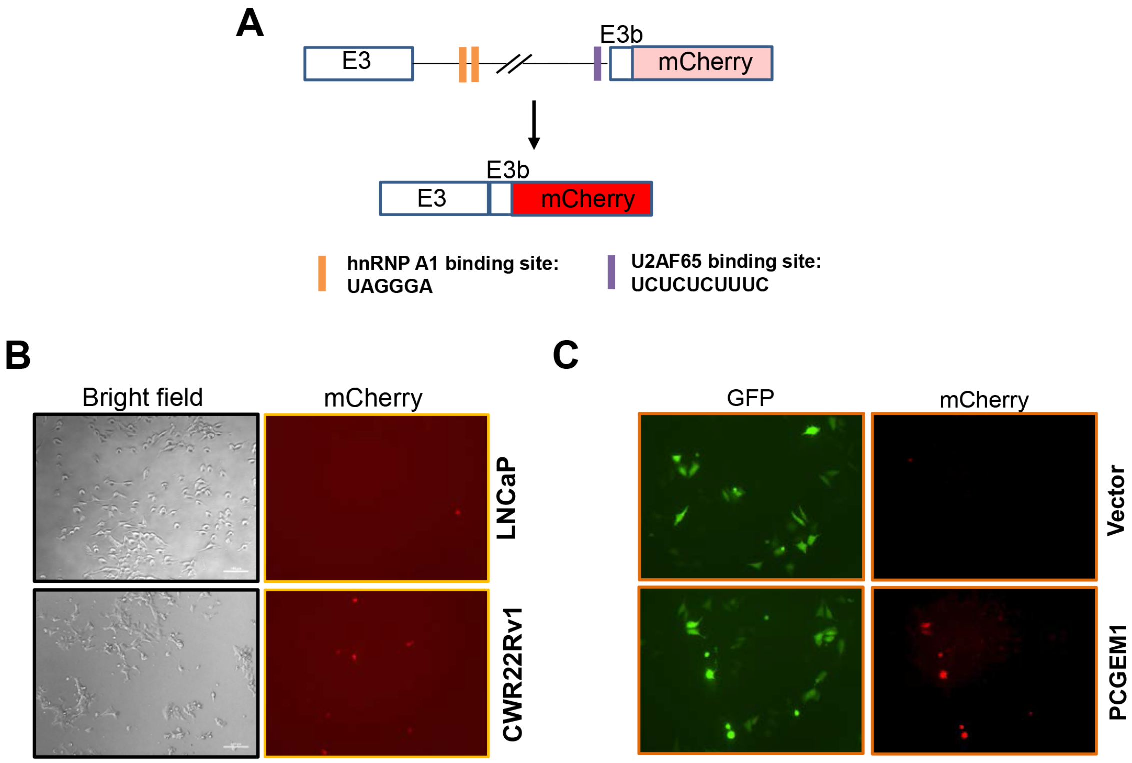

Supplementary Figure S5: Regulation of AR3 expression as determined by mCherry reporter. A. AR3 mini gene cassette. Exonization of E3b leads to expression of fusion protein carrying part of AR3 and mCherry. **B.** The mCherry signal is higher in CWR22Rv1 than in LNCaP cells. **C.** Ectopic expression of PCGEM1 (the vector also carries GFP) increases the mCherry signal compared to vector control.

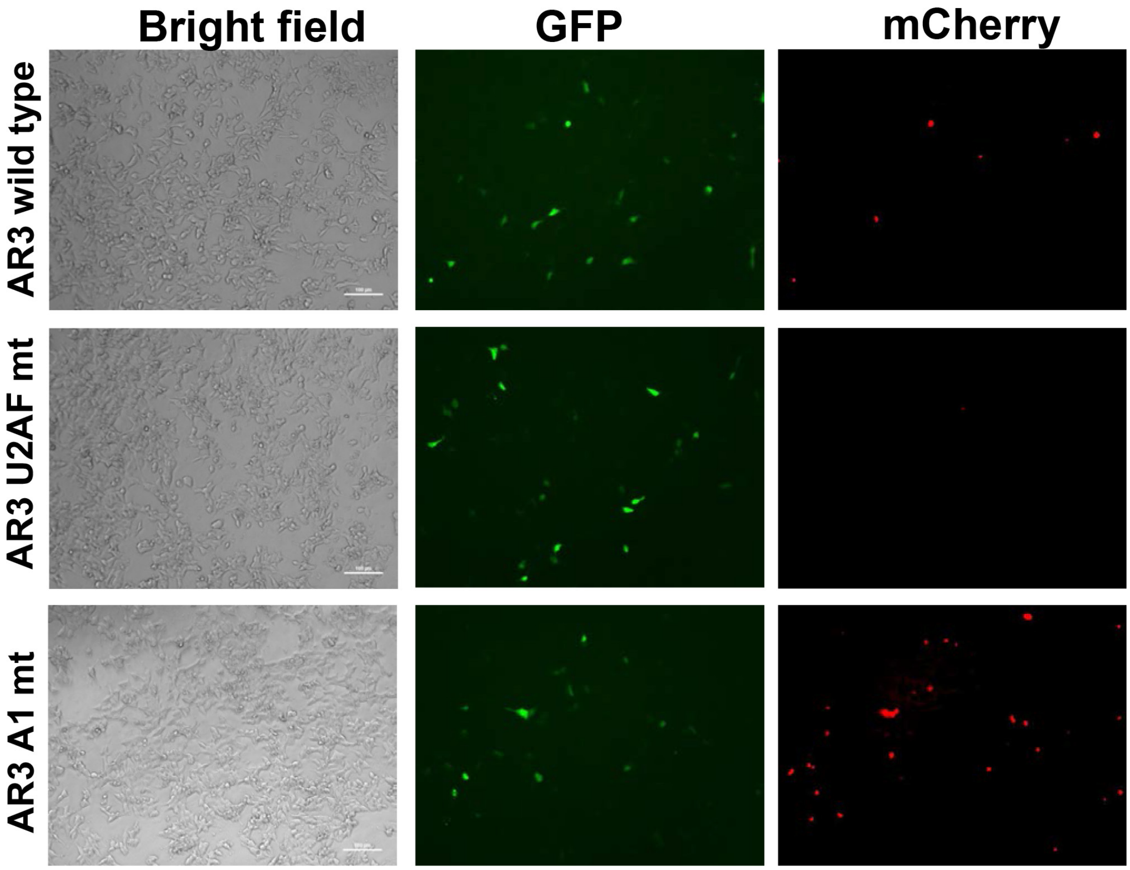

Supplementary Figure S6: Binding sites for hnRNP A1 and U2AF65 in AR pre-mRNA are important to AR3 expression. CWR22Rv1 cells were transfected with mini-gene cassette reporters as indicated and images were taken 24 h after transfection. GFP serves as a transfection control.

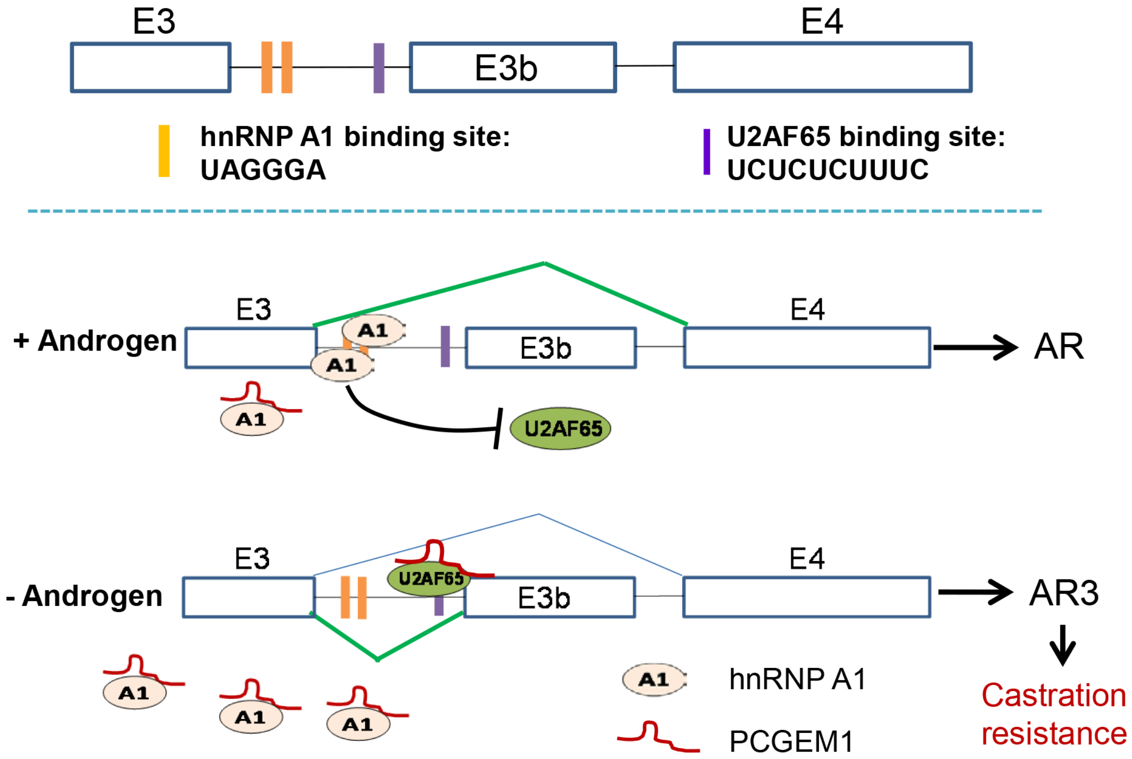

Supplementary Figure S7: A working model of PCGEM1-mediated AR3 in response to androgen deprivation through interaction with splicing factors hnRNP A1 and U2AF65. See text for detail explanation.

Supplementary Table 1. Oligonucleotides used in the study

See Supplementary File 1
