# Supplementary material for: Regulation of androgen receptor splice variant AR3 by PCGEM1
Source: Oncotarget. 2016 Feb 2;7(13):15481–91. doi: 10.18632/oncotarget.7139 (PMC4941255; doi:10.18632/oncotarget.7139)
Supplement: Supplementary file 2 [file oncotarget-07-15481-s002.docx]

Supplementary Table 1. Oligonucleotides used in the study

**For detection by RT-PCR**

To detect full length AR

AR-RT-5.1 Gacgcttctaccagctcacc

AR-RT-3.1 Gcttcactgggtgtggaaat

AR-RT-5.3 accgaggagctttccagaat

AR-RT-3.3 ctggggctagtctcttgctg

To detect AR3

AR3-RT-5.1 gcaattgcaagcatctcaaa

AR3-RT-3.1 ggagggagtcagcaatcaag

To detect AR pre-mRNA

AR-I3-RT-A1-5.1 gactggggtcagggagtaca

AR-I3-RT-A1-3.1 agatggcaagccaagaggtt

Covers A1 binding sites

AR-I3-RT-U2AF-5.1 ccccaactttacatgctgct

AR-I3-RT-U2AF-3.1 GTTTCTCCAGACTATCCACT

Covers U2AF65 binding site

**To detect PCGEM1**

PCGEM1-RT-5.1 Tttttgccctatgccgtaac

PCGEM1-RT-3.1 ggagactcccaacctgatga

PCGEM1-RT-5.2 Ggtaggcacgtggaggacta

PCGEM1-RT-3.2 tgcttttgtgggtttgttca

**To clone PCGEM1**

PCGEM1-MSCV-R1-5.1

ttCtagagctagcgaattcaggcactctggcacccagtt

pCGEM1-MSCV-Not1-3.1 TcgcagatccttgcggccgcgaaatctttaatatttgttA

**For RNA precipitation**

T7-PCGEM1-5.1 TAATACGACTCACTATAGGGaggcactctggcacccagtt

pCGEM1-Not1-3.1 GCGGCCGCgaaatctttaatatttgttA

**For in situ hybridization**

PCGEM1-LNA-1

/5Biosg/t+t+t+tccaaaggg+t+ccgctgtccctg+g+a+g

PCGEM1-LNA-2

/5Biosg/A+t+t+cccctcaga+a+atctcagggctt+g+t+c

PCGEM1-blocker-1 Ctccagggacagcggaccctttggaaaa

PCGEM1-blocker-2 Gacaagccctgagatttctgaggggaat

**To make AR3 mini gene cassette**

pCDH-Myc-R1-AR3-E3-5.1 CCATGGAGGCCCGAATTCtggGGAAACAGAAGTACCTGTGC

AR-I3-5.2+I3-3.1-R

ctgggtggctgcgtgttttTtaaactagatctgcctgact

AR-I3-5.2+I3-3.1-F

agtcaggcagatctagtttaAaaaacacgcagccacccag

AR3-mC-5.1

TGACTTGCCTCATTCAAAAgTGGTGAGCAAGGGCGAGGAG

AR3-mC-3.1

CTCCTCGCCCTTGCTCACCAcTTTTGAATGAGGCAAGTCA

mC-pCDH-Sal1-3.1

tccagaggttgattgtcgacTTACAGCTCGTCCATGCCGC

AR3-I3-RV-5.1 taatatttctgtgacaacag

AR3-mC-5.1 TGACTTGCCTCATTCAAAAgTGGTGAGCAAGGGCGAGGAG

AR3-mC-3.1 CTCCTCGCCCTTGCTCACCAcTTTTGAATGAGGCAAGTCA

AR-I3-5.2 Cttcgatttggaaatggaatttgaagaaaggcaagcctat

AR-I3-3.2 ataggcttgcctttcttcaaattccatttccaaatcgaaG

AR3-I3-Not1-3.2 GCGGCCGCggggctgcaacattcatatg

AR3-I3-Not1-3.1 GCGGCCGCcaaatgttatgaggactttt

**To mutate two internal ATG of mCherry**

mC-mut-5.1 GGCGAGGAGGATAACATAGCCATCATCAAGGAGTTCATACGCTTCAAGGTGCACA

mC-mut-3.1 TGTGCACCTTGAAGCGTATGAACTCCTTGATGATGGCTATGTTATCCTCCTCGCC

AR-I3-Bsu36-5.1 gaaagagactgatgact

mC-Sbf1-3.1 CCTTGTAGATGAACTCGCCG

**To make A1 and U2AF65 binding site mutations**

A1-mt-5.1 ttatgtatagtataaaagaatgcctctctcaagatgcctacctcttggcttgccatct

A1-mt-3.1 agatggcaagccaagaggtaggcatcttgagagaggcattcttttatactatacataa

AR-E3-R1-5.1 GGCCATGGAGGCCCGAATTC

AR-I3-BsrG1-3.1 cctagcatggtgccttgtaca

AR-ISE-mt-5.1 gactgctacagtcaacaatgAcAcAcAAAcaGactagAAAAATTCCGGGTTG

AR-ISE-mt-3.1 CAACCCGGAATTTTTctagtCtgTTTgTgTgTcattgttgactgtagcagtc

**To make hnRNP A1 knockout**

A1-dual-T1-5.1 CAGCAGCATCTTACCGATTC

A1-dual-T2-3.1 GATACCATACCTTGGTTTCG

A1-right-R1-5.2 TTATACGAAGTTATGAATTCCCAAGgtatggtatctatgt

A1-right-R1-3.2 ATAAGCTTGATATCGAATTCAACCTGTTATACAATTAGTC

A1-left-BamH1-5.1 GCTCTAGAACTAGTGGATCCTTACCCGGGATTGAGAGTGA

A1-left-BamH1-3.1 GCTATACGAAGTAGGGATCCAAGAGAGACTTTAACGATGC

| \| siRNAs \| \| --- \| |
| --- | --- |
| \|  \|  \| \| --- \| --- \| |

PCGEM1-siRNA-1(from IDT)

rGrUrGrGrCrArArCrArGrGrCrArArGrCrArGrArGrGrGrAAA

PCGEM1-siRNA-2 (from IDT)

rArGrArCrUrArUrGrArArGrGrUrCrArUrArArArUrUrCrAAC

pCGEM1-siRNA-3 (from Dharmacon)

CAGCCAAAGUGGAACUAAAdTdT

A1 winner

UAUGAUAGGGACUUAGGGUG
